# Elevated gamma-glutamyl transpeptidase level is associated with an increased risk of hip fracture in postmenopausal women

**DOI:** 10.1038/s41598-022-18453-9

**Published:** 2022-08-17

**Authors:** Kyoung Jin Kim, Namki Hong, Min Heui Yu, Seunghyun Lee, Sungjae Shin, Sin Gon Kim, Yumie Rhee

**Affiliations:** 1 Division of Endocrinology and Metabolism, Department of Internal Medicine, Korea University College of Medicine, 73 Inchon-ro, Seongbuk-gu, Seoul, 02841 Republic of Korea; 2 Department of Internal Medicine, Severance Hospital, Endocrine Research Institute, Yonsei University College of Medicine, 50-1 Yonsei-ro, Seodaemun-gu, Seoul, 03722 Republic of Korea; 3 SENTINEL Team, Division of Endocrinology, Department of Internal Medicine, Yonsei University College of Medicine, 50-1 Yonsei-ro, Seodaemun-gu, Seoul, 03722 Republic of Korea

**Keywords:** Biomarkers, Endocrinology

## Abstract

The aim of this study was to evaluate the association between gamma-glutamyl transferase (GGT) levels and the risk of hip fracture among middle-aged women by using the Korean National Health Insurance Service claims database from 2002 to 2015. After exclusion of those with any chronic liver disease, heavy alcohol consumption, any missing values required for our analysis, or GGT levels less than 1 or greater than 99 percentile, we classified subjects into three groups according to baseline GGT levels. A total of 127,141 women aged 50 years or older were included for analysis (GGT range: 8–106 U/L). During an average 12.1 years of follow-up, 2758 patients sustained hip fractures (2.17%). Compared with the group in the lowest tertile, the group in the highest tertile had the highest cumulative incidence of hip fracture. One log-unit increase in GGT was associated with a 17% increased risk of hip fracture. Subgroup analysis by BMI (≥ 25 vs. < 25 kg/m^2^), presence of diabetes, levels of other liver enzymes, and alcohol consumption level did not show significant effect modification. In summary, elevated baseline GGT level was associated with an increased risk of hip fracture in postmenopausal women, independent of alcohol consumption and chronic liver disease.

## Introduction

Hip fracture is a major health concern with rising levels of morbidity and mortality due to rapidly aging populations, imposing a large economic burden on many countries^1,2^. Globally, the number of patients suffering from hip fracture is expected to rise from 1.7 million in 1990 to 6.3 million in 2050^3^. South Korea, for instance, is becoming a super-aged society, and has seen dramatic increases in the incidence of hip fractures^4^. Approximately 40% of patients with hip fracture experience complications, and there is a 5- to 8-fold higher risk of all-cause mortality within the first 3 months after the event^5,6^. Therefore, identifying potential biomarkers to predict the risk of hip fracture is essential to help reduce this burden. Although bone mineral density (BMD) measurements and a fracture risk assessment tool have substantially contributed to the prediction of osteoporotic fractures, many patients at high risk of incident hip fracture are still not identified^7^. In this regard, previous epidemiologic studies have reported that elevated gamma-glutamyl transferase (GGT) activity could be a possible marker for a high risk of low bone mineral density and fractures^8–13^.

In clinical practice, increased circulating GGT levels are routinely associated with potential hepatic or biliary disease and interpreted as a biological marker of excess alcohol intake^14^. Beyond its relationship with liver disease and alcohol, previous studies have suggested that circulating serum GGT is associated with several adverse outcomes, including cardiovascular disease, metabolic syndrome, stroke, and fracture, although the underlying mechanisms linking GGT with these diseases remain to be elucidated^14,15^. In support of the above findings, GGT plays an important role in oxidative stress and inflammation, which can also affect bone metabolism^15,16^. However, more evidence with the large-scale population-based cohort database for a period long enough to observe fracture outcomes is needed to clarify the relationship between serum GGT levels and the risk of hip fracture beyond liver damage, especially among middle-aged to older women at high risk of fracture.

Therefore, the aim of this study was to evaluate the association between serum GGT levels and the risk of hip fracture among postmenopausal women whose GGT would be not that high level after excluding those with liver disease and heavy alcohol consumption. We hypothesized that postmenopausal women with elevated GGT levels would have a higher risk of hip fracture.

## Methods

### Data sources

We used the Korean National Health Insurance Service-Health Screening Cohort (NHIS-HEALS) database to obtain data from January 2002 to December 2014. This database included 514,866 individuals aged between 40 and 79 years in 2002, and participants were followed up until 2015. This comprised 10% of a random selection of the total number of health screening participants between 2002 and 2003. The NHIS requires all insured employees and self-employed persons, as well as their dependents, to undertake a general health screening biannually to improve the health status of Koreans through the prevention and early detection of diseases. During the follow-up period, the cohort was refreshed annually by adding a representative sample once a patient dies to maintain the 10% sampling rate. This database includes individual socio-demographic information and medical and pharmaceutical information such as disease code records according to the International Classification of Disease, Tenth Revision (ICD-10), medical procedures, hospitalization, information on prescribed drugs, and death records. Results of regular laboratory tests, including assessment of GGT levels, were also collected. The detailed cohort protocol was described previously and a growing body of evidence indicates that this cohort is a useful source of data with high generalizability in the Korean Population^17,18^.

This study was conducted in accordance with the declaration of Helsinki. All research procedures and ethical aspects were approved by the institutional review board of Severance Hospital (IRB number: 4-2020-0297).

### Study population

**Figure 1:**
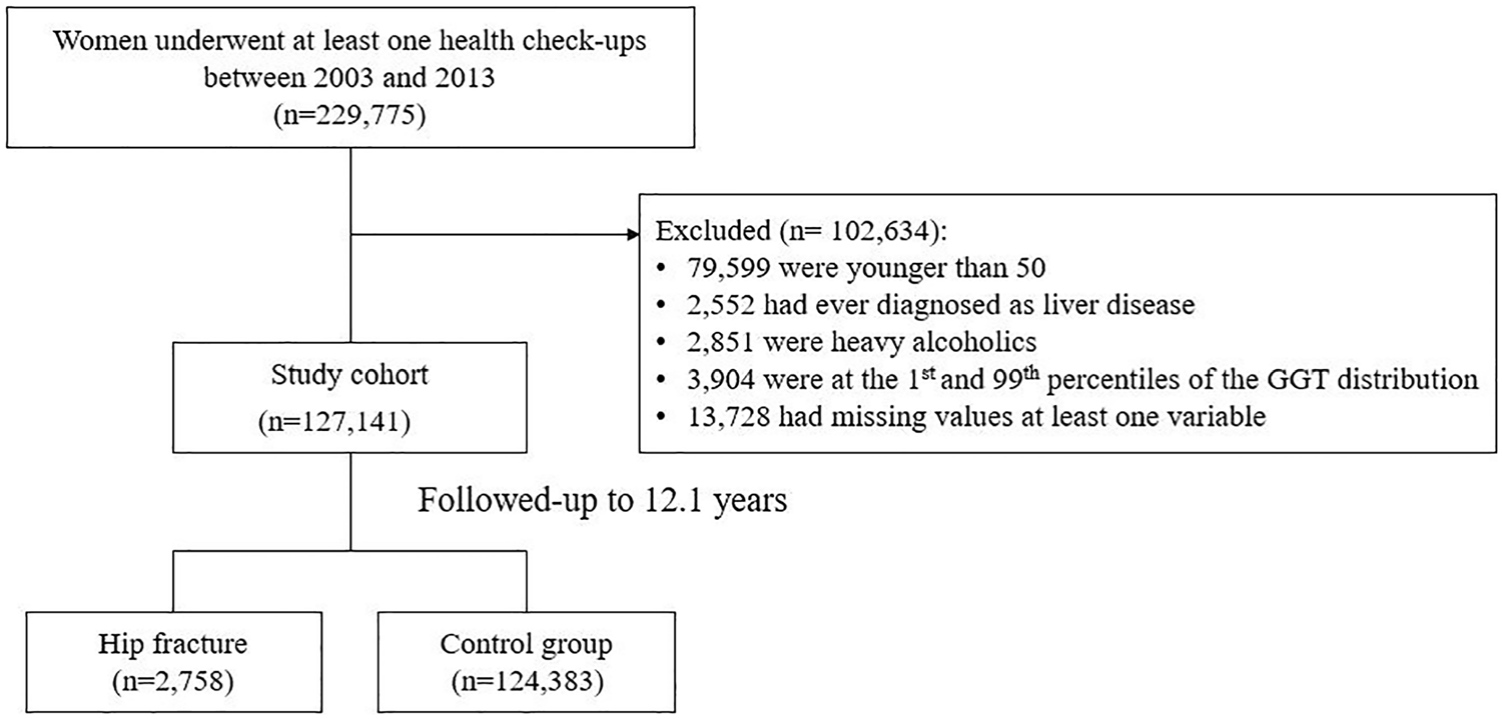
Flow diagram of study participants.

Figure 1 shows the flow diagram of study participants. The present study included female participants who underwent a general health examination at least once between January 2003 and December 2013, after a 1-year washout period. Among the 229,775 women, patients who were younger than 50 years, had any previous history of liver disease (liver cirrhosis, chronic hepatitis, or hepatobiliary malignancy), were heavy alcohol consumers (average daily alcohol intake ≥ 30 g), or had any missing values required for our analysis were excluded. We also excluded subjects whose GGT levels were extremely low or high (in the bottom or top 1%). Ultimately, 127,141 subjects were included in our analysis.

### Study outcomes

Hip fracture developed after the general health examination was included as the main endpoint of our study. We identified patients with hip fractures using the diagnostic codes with ICD-10 (S72.0 and S72.1) and the procedure codes related to hip fractures (N0601, N0991, N0981, N0641, N0652, N0654, N0715, N0711, N0611, N2070, N2710)^19^. Diagnostic codes had to be claimed within the first year prior to procedure codes, in cases where the index date was the date the procedure was performed. We also considered patients who had been hospitalized through the emergency room with the abovementioned procedure codes as cases, where the index date was the date of the patient’s visit to the emergency room. Each participant was followed-up from the index date to the earliest occurrence of hip fracture, death, or end of the study period (31 December 2015).

### Covariates

Covariates included GGT as the exposure variable, age, body mass index (BMI), systolic blood pressure, alcohol consumption (none, or less than 30 g/day), smoking status (current, ever, or never), physical activity (none, ≤ twice per week, or ≥ three times per week), socioeconomic status (SES) (lowest 30%, middle 40%, or highest 30%), aspartate transaminase (AST) level, alanine transaminase (ALT) level, fasting blood glucose level, total cholesterol level, hemoglobin, comorbidities (previous osteoporotic fracture, osteoporosis, rheumatoid arthritis, diabetes mellitus, hypertension, and dyslipidemia), and concurrent medications (glucocorticoid, rifampin, and anticonvulsants). Comorbidities were defined as relevant claim codes during the period ranging from 1 year prior to the cohort entry date to the index date. Concurrent medication was generally considered when patients had taken a prescription within 6 months before to within 2 months after the index date. The details are summarized in Supplemental Table 1.

### Statistical analysis

Participants were classified into three groups according to baseline GGT levels (first tertile [T1], ≤ 14; second tertile [T2] 15–21; and third tertile [T3] ≥ 22 U/L). The baseline characteristics are presented as mean ± SD for continuous variables with normal distribution and median with interquartile ranges for continuous variables with non-normal distribution. Categorical variables are expressed as numbers with percentages. One-way analysis of variance and the chi-square test were used to evaluate the significance of the differences in baseline characteristics between GGT tertiles. The cumulative incidence of hip fracture according to tertiles of baseline GGT was analyzed using Kaplan–Meier estimates, and the log-rank test was performed to compare differences among groups. Furthermore, multivariable Cox regression models were used to analyze the effects of elevated GGT on hip fracture risk considering relevant covariates. Model 1 was adjusted for age and BMI, model 2 was additionally adjusted for alcohol consumption, current smoking, regular exercise, SES, comorbidities, and concurrent medication, and model 3 was further adjusted for laboratory findings including AST, ALT, and hemoglobin. In addition, subgroup analyses were conducted for age (≥ 65 and < 65 years), BMI (≥ 25 and < 25 kg/m^2^), presence of preexisting diabetes mellitus, presence of any abnormal results in AST or ALT, and alcohol consumption (moderate and non-drinker), where GGT values were log-transformed.

Statistical significance was defined as a two-sided p-value < 0.05. All analyses were performed using SAS software (version 9.4; SAS Institute Inc., Cary, NC, USA).

### Ethics approval

Informed consent was waived because data from the NHIS-HEALS did not contain any personally identifiable data.

## Results

### Baseline characteristics of study populations

The mean age and BMI of the study participants were 60.5 ± 7.7 years and 24.3 ± 3.1 kg/m^2^, respectively. The minimum and maximum values of serum GGT were 8–106 U/L for all participants. Table 1 presents the demographic and clinical characteristics of the subjects according to tertiles of baseline GGT levels. The highest tertile group (tertile 3) had more unhealthy factors such as higher BMI, systolic blood pressure, and prevalence of moderate consumers of alcohol and current smokers, and lower SES compared to the lowest tertile group (tertile 1). In addition, the following factors were identified as being significantly correlated with GGT in the present study: AST, ALT, fasting glucose, total cholesterol, and hemoglobin. Patients in the highest GGT tertile also tended to have more comorbidities, including rheumatoid arthritis, diabetes mellitus, hypertension, and dyslipidemia, than patients in the lowest tertile. In addition, glucocorticoid, rifampin, and anticonvulsants were more frequently used in the highest GGT tertile group.

**Table 1:** Baseline characteristics according to tertiles of serum GGT levels.

Characteristics	Tertiles of GGT among women (n = 127,141)			
	T1 (GGT ≤ 14 U/L) (N = 41,711)	T2 (GGT 15–21 U/L) (N = 42,044)	T3 (GGT ≥ 22U/L) (N = 43,386)	p-value
GGT (U/L)	11.44 (11.42–11.46)	17.54 (17.53–17.56)	32.79 (32.10–32.28)	< 0.001
Age (years)	60.46 ± 7.97	60.69 ± 7.66*	60.48 ± 7.45^†^	< 0.001
Body mass index (kg/m^2^)	23.56 ± 2.91	24.27 ± 3.06*	25.08 ± 3.20*^†^	< 0.001
Systolic blood pressure (mmHg)	126.88 ± 18.74	128.96 ± 18.73*	131.12 ± 18.89*^†^	< 0.001
Alcohol consumption, *n* (%)-moderate	3810 (9.13)	4462 (10.61)*	5711 (13.16)*^†^	< 0.001
**Smoking status, *n* (%)**				< 0.001
Never	40,559 (97.24)	40,797 (97.03)	41,733 (96.19)*^†^	
Ex-smoker	326 (0.78)	288 (0.68)	345 (0.80)	
Current smoker	826 (1.98)	959 (2.28)*	1308 (3.01)*^†^	
**Physical activity, *n* (%)**				0.055
None	27,315 (65.49)	27,459 (65.31)	28,455 (65.59)	
≤ twice per week	6593 (15.81)	6657 (15.83)	7016 (16.17)	
≥ three times per week	7803 (18.71)	7928 (18.86)	7915 (18.24)	
**Socioeconomic status, *n* (%)**				0.016
Lowest (30%)	11,415 (27.37)	11,516 (27.39)	11,984 (27.62)*^†^	
Middle (40%)	13,862 (33.23)	14,113 (33.57)	14,786 (34.08)*	
Highest (30%)	16,434 (39.40)	16,415 (39.04)	16,616 (38.30)*	
**Laboratory findings**				
AST (U/L)	21.72 (21.68–21.77)	22.88 (22.83–22.93)*	26.42 (26.35–26.50)*^†^	< 0.001
ALT (U/L)	16.16 (16.11–16.20)	18.74 (18.69–18.80)*	24.76 (24.66–24.56)*^†^	< 0.001
Fasting glucose (mg/dL)	94.70 ± 26.21	97.18 ± 30.19*	103.39 ± 36.66*^†^	< 0.001
Total cholesterol (mg/dL)	201.19 ± 35.78	209.18 ± 37.80*	216.26 ± 41.02*^†^	< 0.001
Hemoglobin (g/dL)	12.76 ± 1.08	12.93 ± 1.05*	13.16 ± 1.07*^†^	< 0.001
**Comorbidities, n (%)**				
Previous osteoporotic fracture	1543 (3.70)	1546 (3.68)	1657 (3.82)	0.498
Osteoporosis	2678 (6.42)	2773 (6.60)	2819 (6.50)	0.588
Rheumatoid arthritis	2849 (6.83)	3316 (7.89)*	3627 (8.36)*^†^	< 0.001
Diabetes mellitus	1866 (4.47)	2779 (6.61)*	4729 (10.90)*^†^	< 0.001
Hypertension	8804 (21.11)	11,459 (27.25)*	14,867 (34.27)*^†^	< 0.001
Dyslipidemia	2271 (5.44)	3431 (8.16)*	5117 (11.79)*^†^	< 0.001
**Concurrent medication, n (%)**				
Glucocorticoid	1870 (4.48)	2188 (5.20)*	2561 (5.90)*^†^	< 0.001
Rifampin	32 (0.08)	54 (0.13)	70 (0.16)*	0.002
Anticonvulsant agents	5028 (12.05)	5887(14.00)*	6978(16.08)*^†^	< 0.001

Data are presented as means ± standard deviations, geometric means (95% confidence interval), or numbers (%).

ALT, alanine aminotransferase; AST, aspartate aminotransferase; GGT, gamma-glutamyl transferase; T, tertile.

*p-value < 0.05 vs T1.

^†^p-value < 0.05 vs T2.

The baseline characteristics of our study subjects according to incident hip fracture status are summarized in Supplemental Table 2. Briefly, patients with hip fracture had higher GGT levels than those without hip fracture (19.20 [18.86–19.55] vs. 18.75 [18.70–18.79], *p* = 0.011). Patients with hip fractures were older and more likely to smoke, have a lower SES, present with comorbidities, and use concurrent medication than without hip fractures. They were also less likely to exercise regularly and consume alcohol. Laboratory findings, such as ALT, fasting glucose, and hemoglobin, were different between the two groups. However, the serum AST and total cholesterol levels did not differ significantly between patients with and without hip fractures.

### Associations between baseline GGT level and hip fracture

**Figure 2:**
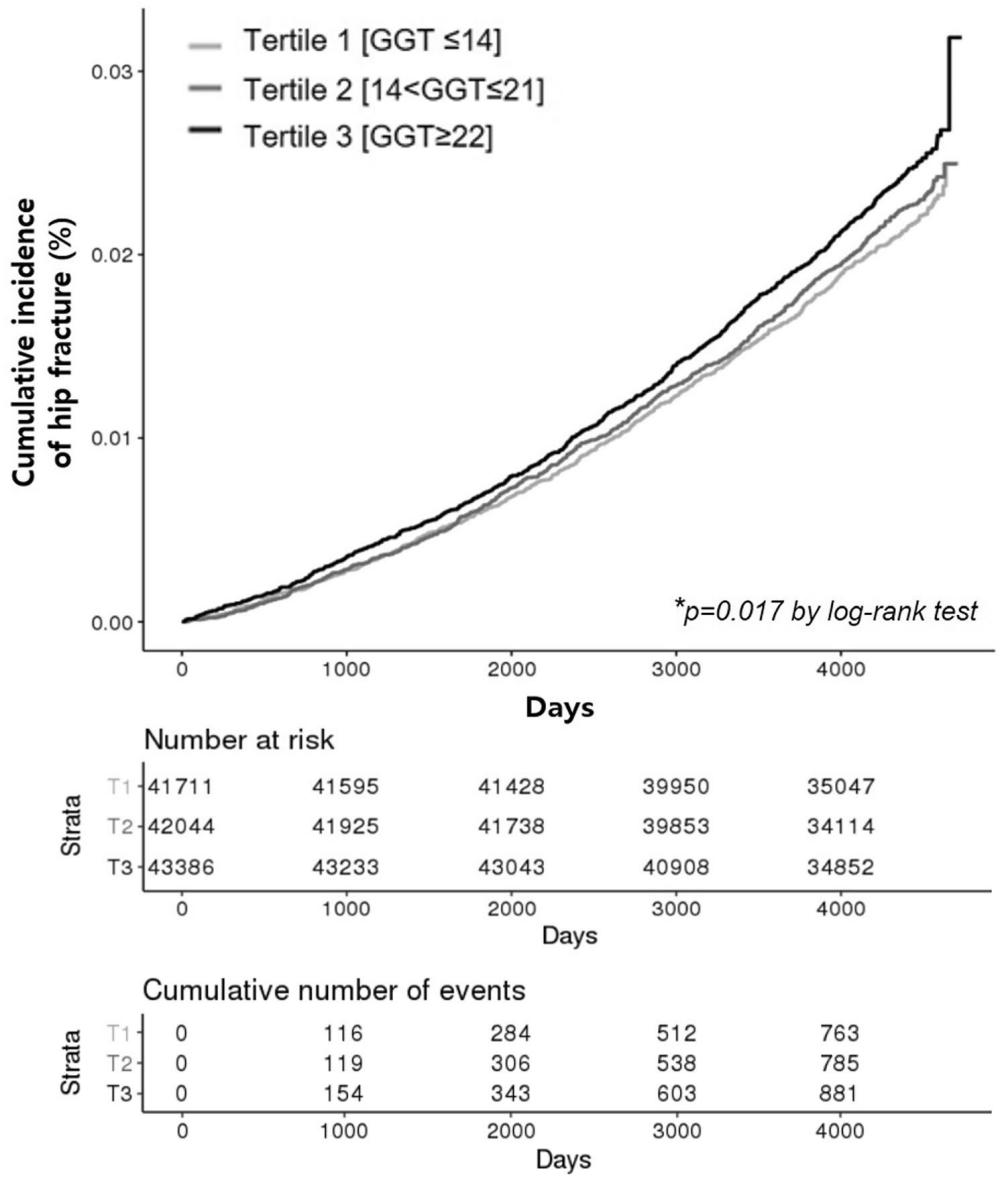
Cumulative incidence of hip fracture according to gamma-glutamyl transferase (GGT) tertiles.

During the median follow-up time of 12.1 years (interquartile range, 11.2–12.4), 2758 participants experienced hip fractures. The incidence of hip fracture increased with increasing tertiles of baseline GGT levels after adjusting for confounding factors (Supplemental Table 3). The hazard ratio (HR) for hip fracture in the highest tertile of baseline GGT levels was 1.16 (95% CI, 1.05–1.28) when compared with the lowest tertile group after fully adjusting for confounding factors. As shown in Fig. 2, Kaplan–Meier estimates of the cumulative incidence of hip fracture also increased progressively with increasing tertiles of baseline GGT during follow-up (log-rank [overall] p = 0.017).

The association between GGT level and risk of hip fracture was assessed using multivariable Cox regression analyses with GGT as exposure (Table 2). An elevated value of logarithmically transformed GGT showed a higher risk of hip fracture, adjusted for other parameters including age, previous history of fracture, osteoporosis, rheumatoid arthritis, diabetes mellitus, hypertension, use of glucocorticoids, and anticonvulsant agents. In this multivariable analysis, the risk of hip fracture increased by 17% with a 1-log increase in GGT level (adjusted HR, 1.17; 95% CI 1.07–1.28). When a sensitivity analysis was performed after the reclassification of subjects into four groups according to the quartiles of GGT (first quartile [Q1], ≤ 13; second quartile [Q2], 14–17, third quartile [Q3], 18–24; and fourth quartile [Q4] ≥ 25 U/L), the results were not markedly different. Women in baseline GGT quartile 4 had an 18% higher risk for hip fracture compared with those in GGT quartile 1 after full adjustment in model 3 (Supplemental Table 4 and Supplemental Fig. 1).

**Table 2:** Multivariable Cox regression analysis of the relative risk of hip fracture.

Factors	Model 1		Model 2		Model 3	
	HR (95% CI)	p-value	HR (95% CI)	p-value	HR (95% CI)	p-value
GGT^a^	1.18 (1.09–1.27)	< 0.001	1.10 (1.02–1.19)	0.019	1.17 (1.07–1.28)	0.001
Age	1.12 (1.11–1.12)	< 0.001	1.11 (1.10–1.12)	< 0.001	1.11 (1.10–1.12)	< 0.001
BMI	1.01 (0.99–1.02)	0.199	1.00 (0.99–1.01)	0.920	1.00 (0.99–1.01)	0.778
Alcohol consumption			0.98 (0.86–1.13)	0.814	0.98 (0.86–1.12)	0.781
Current smoking			1.17 (0.97–1.42)	0.105	1.16 (0.96–1.41)	0.130
Regular exercise			0.97 (0.87–1.07)	0.531	0.97 (0.87–1.08)	0.781
Lowest SES			0.95 (0.87–1.04)	0.280	0.95 (0.87–1.04)	0.289
Previous fracture			1.34 (1.16–1.55)	< 0.001	1.34 (1.16–1.54)	< 0.001
Osteoporosis			1.31 (1.16–1.47)	< 0.001	1.31 (1.16–1.47)	< 0.001
Rheumatoid arthritis			1.40 (1.25–1.57)	< 0.001	1.40 (1.25–1.57)	< 0.001
Diabetes mellitus			1.77 (1.59–1.97)	< 0.001	1.76 (1.58–1.97)	< 0.001
Hypertension			1.14 (1.05–1.24)	0.003	1.14 (1.05–1.24)	< 0.001
Dyslipidemia			0.89 (0.78–1.02)	0.092	0.89 (0.78–1.02)	0.092
Glucocorticoid			1.33 (1.16–1.53)	< 0.001	1.32 (1.15–1.52)	< 0.001
Rifampin			1.06 (0.48–2.37)	0.882	1.07 (0.48–2.39)	0.867
Anticonvulsant agents			1.15 (1.05–1.26)	0.004	1.15 (1.04–1.26)	0.005
AST^a^					0.87 (0.75–1.01)	0.071
ALT^a^					0.92 (0.82–1.04)	0.184
Hemoglobin					0.99 (0.96–1.03)	0.814

Model 1: adjusted for age and BMI; model 2: adjusted for model 1 plus alcohol consumption, current smoking, regular exercise, lowest SES, previous fracture, osteoporosis, rheumatoid arthritis, diabetes mellitus, hypertension, dyslipidemia, and use of glucocorticoid, rifampin, and anticonvulsant agents; model 3: adjusted for model 2 plus laboratory findings of AST, ALT, and hemoglobin.

ALT, alanine aminotransferase; AST, aspartate aminotransferase; BMI, body mass index; CI, confidence interval; GGT, gamma-glutamyl transferase; HR, hazard ratio; SES, socioeconomic status; T, tertile.

^a^GGT, AST and ALT were logarithmically transformed.

### Subgroup analysis

**Figure 3:**
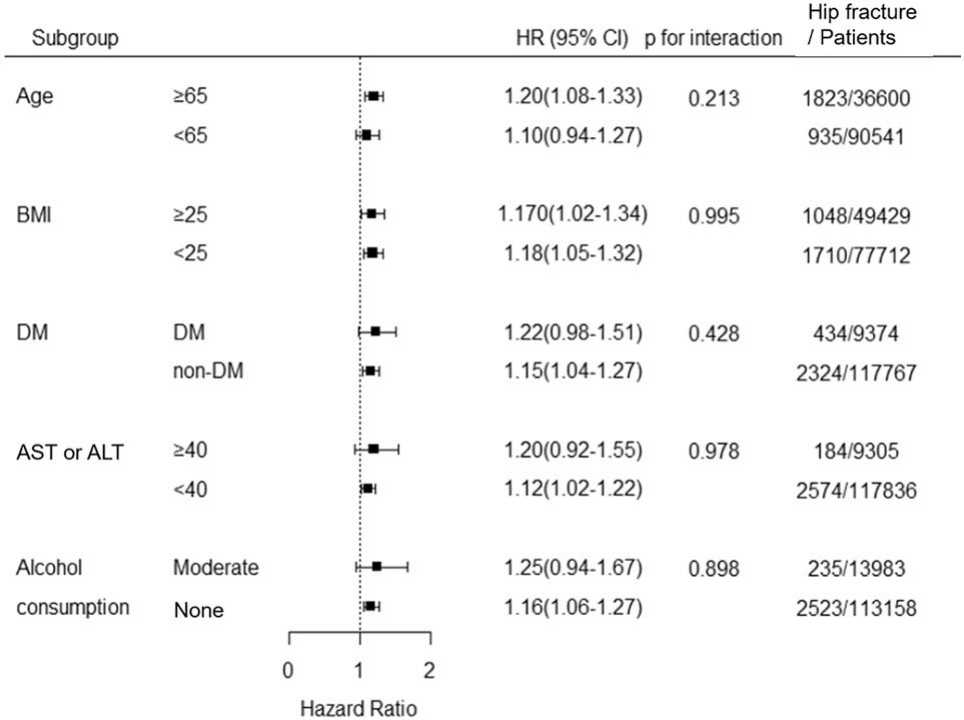
Subgroup analysis with adjusted hazard ratios for the presence of incident hip fracture per single-unit increase in log-gamma-glutamyl transferase (GGT) as a continuous variable. Results have been adjusted for age, BMI, alcohol consumption, smoking history, regular exercise, SES, previous history of fracture, osteoporosis, rheumatoid arthritis, DM, hypertension, dyslipidemia, use of glucocorticoid, rifampin, anticonvulsant agents, and laboratory findings of AST, ALT, and hemoglobin. ALT, alanine aminotransferase; AST, aspartate aminotransferase; BMI, body mass index; CI, confidence interval; DM, diabetes mellitus; GGT, gamma-glutamyl transferase; HR, hazard ratio; SES, socioeconomic status.

Figure 3 shows the results of subgroup analyses of the association between GGT and the risk of hip fracture among middle-aged to older women after stratification by age, BMI, diabetes mellitus, higher levels of AST or ALT, and alcohol consumption. No significant interactions were found between GGT levels and all stratified variables (p for all interactions > 0.05). Higher GGT levels were associated with a higher risk of hip fracture than lower GGT levels in most subgroups.

## Discussion

In this nationwide cohort of middle-aged and older women with no history of chronic liver disease, or heavy alcohol consumption, higher serum GGT concentration was associated with an increased risk of incident hip fracture. The result remained significant after adjusting for multiple variables, including alcohol consumption and fracture risk factors. In addition, the serum GGT, even if the levels were not that high, had a dose–response relationship with incident hip fractures.

GGT is a ubiquitous cell surface glycoprotein known to play a primary role in intracellular antioxidant defense by catabolizing glutathione^14^. In this regard, increased GGT levels could be an early marker for oxidative stress associated with pathological diseases beyond liver damage, including cardiovascular disease, stroke, dementia, diabetes, metabolic syndrome, cancer, and abnormal bone metabolism^14,15,20^. Previous in vitro studies have suggested that oxidative stress directly deteriorates bone metabolism by enhancing osteoclast activity through expression of receptor activator for NF-κB-ligand in marrow stromal cells and inhibiting osteoblast differentiation^21,22^. Additionally, a clinical study suggested that urinary GGT excretion was highly associated with deoxypyridinoline, a well-known bone resorption marker, which established its function as a direct marker of bone resorption^9^. In terms of indirect association, a longitudinal epidemiologic study has also shown that higher GGT levels are associated with increased levels of inflammatory markers such as C-reactive protein and fibrinogen^23^. Furthermore, a recent review pointed out the important influence of iron metabolism in incident fractures and that GGT is also an indirect indicator of iron overload related to oxidative stress^24^. Therefore, increased levels of GGT directly or indirectly induced by excess oxidative stress may reflect its negative effects on bone resulting in osteoporotic fracture.

Thus far, the relationship between baseline GGT concentration and skeletal health, including bone metabolism, has been continuously evaluated. Prospective cohort studies with Swedish middle-aged women and men suggested that elevated GGT above normal range was associated with increased risks for low-trauma fractures and specifically for hip fractures^11,12^. A large prospective study involving 16,036 Korean men over a mean 3-year follow-up period also provided clinical evidence that a higher level of GGT may be an independent risk factor for the detection of incident osteoporotic fractures^10^. Recently, Brozek et al.^13^ confirmed a positive association between elevated GGT concentrations and an increasing risk of future hip fracture. Choi et al.^8^ revealed that serum GGT levels were inversely associated with BMD in a total of 462 subjects. In their study, being in the highest tertile of serum GGT level increased the risk of low bone mass, leading to osteopenia and osteoporosis. Another investigation including 7160 Koreans also demonstrated that GGT had a relatively clear negative association with BMD at multiple sites^25^. A study regarding the association between liver markers and short-term outcomes in patients with hip fracture demonstrated that higher GGT levels at the time of admission increased the risk of a prolonged hospital stay^26^. Hong et al.^27^ further claimed that serum GGT activity was associated with the development of sarcopenia, which is a risk factor for falls and fractures, in 3193 community-dwelling adults. As in previous investigations, our study consistently showed that a high GGT level suggested poor outcomes of bone metabolism followed by hip fractures among middle-aged women.

Several confounding factors have a substantial influence on GGT levels. The most representative factors are sex, liver damage, and excessive consumption of alcohol^15^. Previous epidemiologic studies evaluated GGT levels and bone health in both men and women, although subgroup analysis was performed^8,27^. We included only women because GGT levels are well known to be higher in men than in women^10^. Previous observational studies also adjusted for these important confounding factors, such as the degree of alcohol consumption and laboratory results of other liver function tests^8,10,27^. After adjusting for confounding factors, we excluded patients with GGT levels in the lowest and highest 1% to decrease variability and performed another subgroup analysis. Results of that analysis still showed a positive relationship between GGT and incidence of hip fracture among patients whose AST or ALT levels were within the normal range (< 40 U/L), as well as in non-drinkers. Furthermore, we showed a dose–response relationship between the risk of hip fracture and GGT level, which was not stronger than in other studies.

The major strength of this study was the large number of subjects and hip fracture cases, which could provide sufficient statistical power and minimize selection and recall biases. The additional strength was its careful enrollment of community-dwelling women who were not heavy alcohol consumers and did not have extensive liver damage based on our exclusion criteria. We also removed outliers of GGT to reduce the potential for type 1 errors^28^. Furthermore, we considered various factors that affect bone metabolism, including demographic and clinical characteristics, comorbidities, and concurrent medications. However, the findings of the present study need to be interpreted with caution because of the following limitations. First, as with all epidemiological studies, our results cannot establish a causal relationship between GGT levels and incident hip fracture. However, previous studies have shown similar results. Second, we defined hip fractures using the Korean NHIS claims database, so there might be inaccurate diagnoses. However, to improve the accuracy of diagnosis, our outcomes were limited to hip fractures rather than other major osteoporotic fractures, such as vertebral fractures, which are more likely to be missed due to a higher incidence of silent fractures^29^. Third, data on serum BMD, vitamin D, and calcium levels were not available in the NHIS-HEALS cohort. Therefore, the baseline status of mineral deficiencies could not be considered. Lastly, caution should be taken when generalizing our results to male patients and other populations because our study included only Korean women without conditions related to excess alcohol consumption and liver disease at baseline. However, the use of careful enrollment allowed the creation of a homogeneous population with GGT levels in a range of not-so-high levels and reduced confounding bias.

In conclusion, higher GGT levels were associated with an increased risk of hip fractures in Korean women. There was also a dose-dependent relationship between increased GGT levels without extreme level and hip fracture risk. Further studies are needed to explore the potential mechanism increased GGT levels in the pathogenesis of hip fracture.

## Supplementary Information

Supplementary Information.

## Data Availability

The data set is available through approval and oversight by the Korean National Health Insurance Service. Statistical code: See the “Supplementary Material”.

## Abbreviations

ALT: Alanine transaminase
AST: Aspartate transaminase
BMD: Bone mineral density
BMI: Body mass index
GGT: Gamma-glutamyl transferase
ICD: International Classification of Disease
Q: Quartile
T: Tertile
SES: Socioeconomic status
