# Supplementary material for: Elevated gamma-glutamyl transpeptidase level is associated with an increased risk of hip fracture in postmenopausal women
Source: Sci Rep. 2022 Aug 17;12:13947. doi: 10.1038/s41598-022-18453-9 (PMC9385606; doi:10.1038/s41598-022-18453-9)

**Supplemental Data**

**Elevated gamma-glutamyl transpeptidase level is associated with an increased risk of hip fracture: a nationwide population-based study**

| **Contents** | **Page** |
| --- | --- |
| **Supplemental Table 1.** Definitions and codes used for key conditions, comorbidities, and drug treatments in this study. | 2-3 |
| **Supplemental Table 2.** Baseline characteristics of subjects with and without hip fractures. | 4-5 |
| **Supplemental Table 3.** Hazard ratios and 95% confidence intervals for the incidence of hip fracture according to tertiles of baseline gamma-glutamyl transferase levels. | 6 |
| **Supplemental Table 4.** Hazard ratios and 95% confidence intervals for the incidence of hip fracture according to quartiles of baseline gamma-glutamyl transferase levels. | 7 |
| **Supplemental Figure 1.** Cumulative incidence of hip fracture according to the gamma-glutamyl transferase quartiles. | 8 |

**Supplemental Table 1.** Definitions and codes used for key conditions, comorbidities, and drug treatments in this study.

|  | **Definitions** | **ICD-10 Codes or conditions** |
| --- | --- | --- |
| **Comorbidities** | |  |
| Liver disease | Defined from diagnostic codes identified during the study period | chronic liver disease: ICD-10 K70.3, K74, B18  hepatobiliary malignancy: C22-25 |
| Previous osteoporotic fracture | Defined from diagnostic codes of vertebral fracture plus non-vertebral fracture  at least once | vertebral fracture: ICD-10 S22.0, S22.1, S32.0, S32.7, T080, M48.4, M48.5, M49.5  non-vertebral fracture: hip fracture (ICD-10 S72.0, S72.1, S72.2), humerus fracture (ICD-10 S42.2, S42.3), or distal radius fracture (ICD-10 S52.5, S52.6) |
| Osteoporosis | Defined from prescription codes for any treatment received at least once | ICD-10 M80-M82  Treatment: bisphosphonate (alendronate, etidronate, ibandronate, risedronate, pamidronate, zoledronate), selective estrogen receptor modulator (bazedoxifene, raloxifene), or hormone replacement therapy (allylestrenol, chlormadinone, dienogest, diethylstilbestrol, dydrogesterone, estradiol, estrogen, ethynylestradiol, levonorgestrel, medroxyprogesterone) |
| Rheumatoid arthritis | Defined from a diagnostic code used at least once | ICD-10 M05, M06, M45 |
| Diabetes mellitus | Defined from diagnosis plus treatment (≥ 30 days) at least once | ICD-10 E10-E14  Treatment: various oral antidiabetics (alpha-glucosidase inhibitor, DPP-4 inhibitor, GLP1 agonist, meglitinide, metformin, SGLT2 inhibitor, sulfonylurea, thiazolidinedione) and insulin. |
| Hypertension | Defined from diagnosis plus treatment (≥ 30 days) at least once | ICD-10 I10  Treatment: various antihypertensive agents (ARB, ACE inhibitors, beta blockers, calcium channel blockers, alpha blockers, diuretics, and others) |
| Dyslipidemia | Defined from diagnosis plus treatment (≥ 30 days) at least once | ICD-10 78  Treatment: various lipid lowering agents (statins, fibrates) |
| **Medication (available in South Korea)** | | |
| Glucocorticoid |  | betamethasone, budesonide, deflazacort, dexamethasone, hydrocortisone, methylprednisolone, prednisolone, triamcinolone |
| Rifampin |  | rifampicin |
| Anticonvulsants |  | barbiturates, benzodiazepines, phenobarbital |

Abbreviations: ACE inhibitor, angiotensin-converting-enzyme inhibitor; ARB, angiotensin II receptor antagonist; DPP-4, dipeptidyl peptidase-4; GLP-1, glucagon-like peptide-1; ICD-10, International Classification of Diseases, 10th revision; SGLT2, sodium glucose co-transporter 2

**Supplemental Table 2.** Baseline characteristics of subjects with and without hip fractures.

| Characteristics | Subjects (n=127,141) | | |
| --- | --- | --- | --- |
|  | With hip fractures  (n=2,758) | Without hip fractures (n=124,383) | p-value |
| GGT (U/L)^a^ | 19.20  (18.86-19.55) | 18.75  (18.70-18.79) | 0.011 |
| Age (years) | 67.53±7.45 | 60.39±7.62 | <0.001 |
| Body mass index (kg/m^2^) | 24.24±3.36 | 24.32±3.12 | 0.249 |
| Alcohol consumption, *n* (%)-moderate | 235(8.52) | 13748(11.05) | <0.001 |
| Smoking status, *n* (%) |  |  | <0.001 |
| Never | 2618(94.92) | 120471(96.85) |  |
| Ex-smoker | 28(1.02) | 931(0.75) |  |
| Current smoker | 112(4.06) | 2981(2.40) |  |
| Physical activity, *n* (%) |  |  | <0.001 |
| None | 2027(73.50) | 81202(65.28) |  |
| ≤ twice per week | 308(11.17) | 19958(16.05) |  |
| ≥ three times per week | 423(15.34) | 23223(18.67) |  |
| Socioeconomic status, *n* (%) |  |  |  |
| Lowest (30%) | 781(28.32) | 34134(27.44) | 0.001 |
| Middle (40%) | 839(30.42) | 41922(33.70) |  |
| Highest (30%) | 1138(41.26) | 48327(38.85) |  |
| Systolic blood pressure (mmHg) | 132.9±19.22 | 128.9±18.85 | <0.001 |
| **Laboratory findings** |  |  |  |
| AST (U/L) ^a^ | 23.50  (23.22-23.78) | 23.63  (23.59-23.67) | 0.352 |
| ALT (U/L) ^a^ | 18.84  (18.53-19.16) | 19.65  (19.60-19.70) | <0.001 |
| Fasting glucose (mg/dL) | 106.9±44.52 | 98.30±31.23 | <0.001 |
| Total cholesterol (mg/dL) | 207.9±40.06 | 209.0±38.76 | 0.138 |
| Hemoglobin (g/dL) | 12.87±1.14 | 12.94±1.08 | 0.001 |
| **Comorbidities, n (%)** |  |  |  |
| Previous  osteoporotic fracture | 224 (8.12) | 4522 (3.64) | <0.001 |
| Osteoporosis | 341 (12.36) | 7929 (6.37) | <0.001 |
| Rheumatoid arthritis | 354 (12.84) | 9438 (7.59) | <0.001 |
| Diabetes mellitus | 434 (15.74) | 8940 (7.19) | <0.001 |
| Hypertension | 1115 (40.43) | 34015 (27.35) | <0.001 |
| Dyslipidemia | 268 (9.72) | 10551 (8.48) | 0.022 |
| **Concurrent medication, n (%)** |  |  |  |
| Glucocorticoid A | 227 (8.23) | 6392 (5.14) | <0.001 |
| Rifampin A | 6 (0.22) | 150 (0.12) | 0.150 |
| Anticonvulsant agents A | 581 (21.07) | 17312 (13.92) | <0.001 |

*Fisher’s exact test p-value, ^a^geometric mean (95% confidence interval).

Abbreviations: ALT, alanine aminotransferase; AST, aspartate aminotransferase; CI, confidence interval; GGT, gamma-glutamyl transferase.

**Supplemental Table 3.** Hazard ratios and 95% confidence intervals for the incidence of hip fracture according to tertiles of baseline gamma-glutamyl transferase levels.

| Baseline GGT | Events of  hip fracture (n) | Incidence rate  (per 1000 person-years) | Model 1 | Model 2 | Model 3 |
| --- | --- | --- | --- | --- | --- |
| T1 | 862 | 1.78 | 1 (ref) | 1 (ref) | 1 (ref) |
| T2 | 894 | 1.86 | 1.05 (0.96-1.15) | 1.01 (0.92-1.11). | 1.03 (0.93-1.13) |
| T3 | 1002 | 2.02 | 1.19 (1.08-1.30) | 1.10(1.00-1.20) | 1.15 (1.04-1.12) |
| *P* for trend* |  |  | <0.001 | 0.053 | 0.006 |

* p-value calculated using the Wald test. The GGT tertiles were modeled as continuous variables using Cox regression.

Model 1: adjusted for age and BMI; model 2: adjusted for model 1 plus alcohol consumption, current smoking, regular exercise, lowest SES, previous fracture, osteoporosis, rheumatoid arthritis, diabetes mellitus, hypertension, dyslipidemia, and use of glucocorticoid, rifampin, and anticonvulsant agents; Model 3: adjusted for model 2 plus laboratory findings of AST, ALT, and hemoglobin.

Abbreviations: GGT, gamma-glutamyl transferase; T, tertile.

**Supplemental Table 4.** Hazard ratios and 95% confidence intervals for the incidence of hip fractures according to quartiles of baseline gamma-glutamyl transferase levels.

| Baseline GGT | Events of  hip fracture (n) | Incidence rate  (per 1000 person-years) | Model 1 | Model 2 | Model 3 |
| --- | --- | --- | --- | --- | --- |
| Q1 (≤13 U/L) | 710 | 1.80 | 1 (ref) | 1 (ref) | 1 (ref) |
| Q2 (14~17 U/L) | 582 | 1.77 | 0.99(0.89-1.11) | 0.96(0.86-1.07) | 0.97(0.87-1.08) |
| Q3 (18~24 U/L) | 699 | 1.93 | 1.10(0.99-1.22) | 1.04(0.94-1.16) | 1.07(0.96-1.19) |
| Q4 (25≥U/L) | 767 | 2.05 | 1.21(1.09-1.34) | 1.10(0.99-1.23) | 1.18(1.05-1.32) |
| *P* for trend* |  |  | <0.001 | 0.029 | 0.002 |

* p-value calculated using the Wald test. The GGT quartiles were modeled as continuous variables using Cox regression.

Model 1: adjusted for age and BMI; model 2: adjusted for model 1 plus alcohol consumption, current smoking, regular exercise, lowest SES, previous fracture, osteoporosis, rheumatoid arthritis, diabetes mellitus, hypertension, dyslipidemia, and use of glucocorticoid, rifampin, and anticonvulsant agents; Model 3: adjusted for model 2 plus laboratory findings of AST, ALT, and hemoglobin.

Abbreviation: GGT, gamma-glutamyl transferase; Q, quartile.

**Supplemental Figure 1.** Cumulative incidence of hip fracture according to gamma-glutamyl transferase quartiles (Q, quartile)


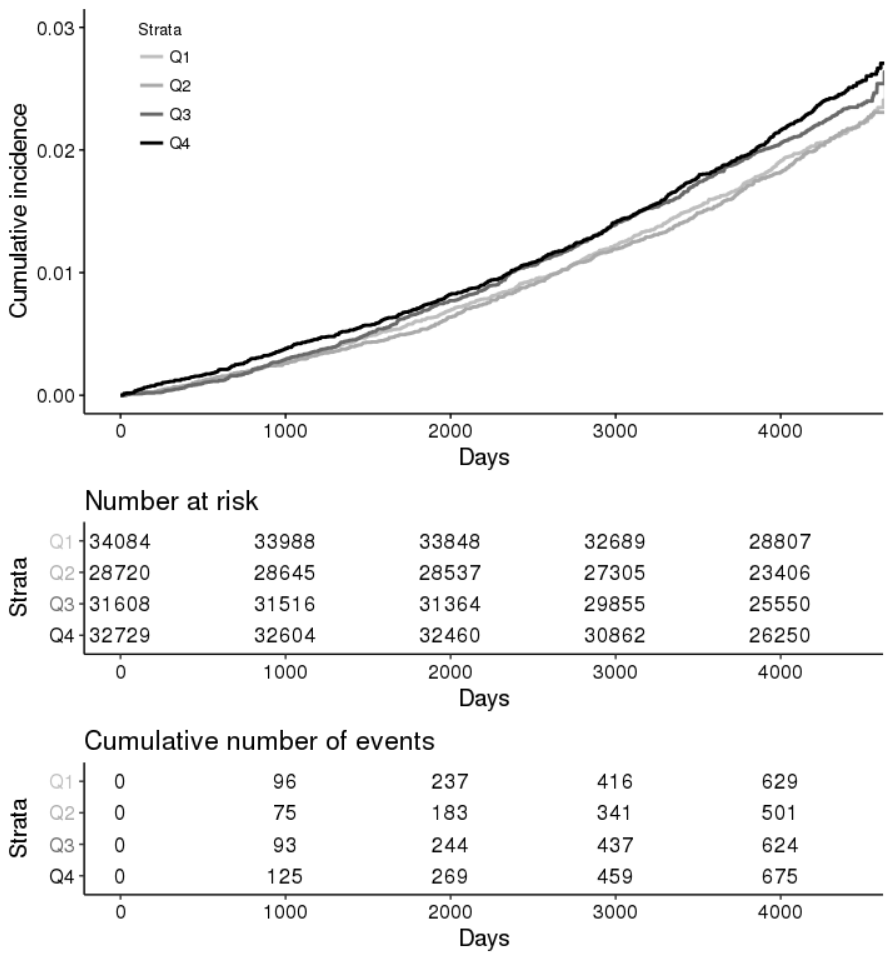

Supplement: Supplementary file 1 — Supplementary Information. [file 41598_2022_18453_MOESM1_ESM.docx]
